# Validated and Predictive Processing of Gas Chromatography-Mass Spectrometry Based Metabolomics Data for Large Scale Screening Studies, Diagnostics and Metabolite Pattern Verification

**DOI:** 10.3390/metabo2040796

**Published:** 2012-10-31

**Authors:** Elin Thysell, Elin Chorell, Michael B. Svensson, Pär Jonsson, Henrik Antti

**Affiliations:** 1 Department of Chemistry, Computational Life Science Cluster (CLiC), Umeå University, SE-901 87 Umeå, Sweden; Email: elin.thysell@chem.umu.se (E.T.); 2 Department of Public Health and Clinical Medicine, Umeå University, SE-901 87 Umeå, Sweden; Email: elin.chorell@medicine.umu.se (E.C.); 3 Department of Surgical and Perioperative Sciences, Sports Medicine, Umeå University, SE-901 87 Umeå, Sweden; Email: michael.svensson@idrott.umu.se (M.S.)

**Keywords:** metabolomics, chemometrics, information, large data, GC/MS, curve resolution, diagnosis

## Abstract

The suggested approach makes it feasible to screen large metabolomics data, sample sets with retained data quality or to retrieve significant metabolic information from small sample sets that can be verified over multiple studies. Hierarchical multivariate curve resolution (H-MCR), followed by orthogonal partial least squares discriminant analysis (OPLS-DA) was used for processing and classification of gas chromatography/time of flight mass spectrometry (GC/TOFMS) data characterizing human serum samples collected in a study of strenuous physical exercise. The efficiency of predictive H-MCR processing of representative sample subsets, selected by chemometric approaches, for generating high quality data was proven. Extensive model validation by means of cross-validation and external predictions verified the robustness of the extracted metabolite patterns in the data. Comparisons of extracted metabolite patterns between models emphasized the reliability of the methodology in a biological information context. Furthermore, the high predictive power in longitudinal data provided proof for the potential use in clinical diagnosis. Finally, the predictive metabolite pattern was interpreted physiologically, highlighting the biological relevance of the diagnostic pattern.

## Abbreviations

CV: Cross validation or Cross validated
GC/TOFMS: Gas chromatography time of flight mass spectrometry
H-MCR: Hierarchical multivariate curve resolution
MCR: Multivariate curve resolution
OPLS: Orthogonal projections to latent structures
OPLS-DA: Orthogonal projections to latent structures discriminant analysis
OSC: Orthogonal signal correction
PCA: Principal component analysis
PLS: Partial least squares projections to latent structures
UPLC/MS: Ultra performance liquid chromatography mass spectrometry

## 1. Introduction

One issue of major concern for the progression of metabolomics [1,2] is efficient screening of large sample sets or sample banks for patterns of co-regulated entities (metabolites) associated with some defined physiological status [3,4,5,6]. With the main aims of improving statistical power and being able to carry out metabolomics-based epidemiological studies, it is seen today as a key objective in analyzing data sets composed of thousands of samples. Nevertheless, this will require reproducible analyses over long time scales, as well as sophisticated and efficient strategies for processing the acquired data to be able to retrieve relevant metabolite information. So far, promising results have been presented using NMR in metabolome-wide associations (MWAS)[7,8] and mass spectrometry in large-scale, non-targeted studies using quality control samples as a means for generating reference tables of putative metabolites, as well as correcting for analytical drifts in the data [9,10]. Another interesting approach that has attracted great interest recently in mass spectrometry-based metabolomics is the use of array-based detection and quantification of pre-defined sets of metabolites. This has been shown to work well in large-scale association studies and is definitely providing a useful complement to non-targeted approaches [11,12,13]. However, despite being of high importance for the progress of the metabolomics field, the main objective of these studies has not been the data processing part with the aim of generating a pipeline for retrieving high quality data.

Mining of sample banks is becoming increasingly important in trying to understand the complex biological interactions behind, or finding diagnostic or prognostic biomarkers for, various disease states. Usually, these sample banks contain a large number of human samples collected continuously over a long period of time, often extremely well-characterized in terms of property data (metadata). Samples of this type are very attractive for research purposes. However, a problem is that regulations regarding availability, for obvious and valid reasons, are very strict, and also, sample volumes might be limited for specific applications. For this reason, it will be of high relevance to be able to select a representative subset of samples for analysis and method development. A way to address this would be to select samples based on the metadata characterization to make sure to create a sample set consisting of samples relevant for the question. The selected sample set could then be characterized using an appropriate analytical technique, and the acquired data processed to obtain a reliable quantification and identification of detected metabolites, *i.e.,* a reference table of putative metabolites in the analyzed samples. In that way, multiple sample comparisons and biomarker or biomarker pattern extraction can be efficiently carried out by means of multivariate data analysis. Verification of the findings in independent sample sets, in the case of one or a few detected selective biomarkers, could be carried out by setting up biological assays for the detected metabolites. However, in the case of using a metabolite pattern or profile as the indicator of a specific physiological state, the data processing and analysis must work in a predictive way so that this pattern or profile can be verified in new samples, and thus work as a diagnostic tool. ‘Predictive’ in this case means a processing algorithm that can efficiently detect and quantify metabolites in the generated reference table in independently analyzed samples. 

To obtain an efficient screening of large sample sets where the aim is to acquire data for all samples, the key issue will be the data processing step. A sophisticated processing of GC/MS data, such as curve resolution, [14,15,16] is time-consuming, which makes it not feasible to process large sample sets. However, the benefits of such a data processing that can provide a reliable metabolite quantification and identification for further sample comparison and biological interpretation do present an incentive to solve this problem. One way of doing this could be to use a fast and crude data processing technique that still retains the variation in the data and then based on that data, select a representative subset of samples for the more sophisticated processing, *i.e.,* generation of a reference table of putative metabolites. Again, a key here is for the sophisticated processing to work predictively for new samples. If this is the case, then the samples not selected for processing, as well as additional samples measured at a later point in time, can be predictively processed to detect and quantify the metabolites in the reference table. GC/MS has proven to be a valuable tool for the global detection of metabolites in biofluids and tissues [17,18,19,20]. This is mainly due to the combination of high sensitivity and reproducibility, but is also due to the fact that identification of detected compounds is relatively straightforward. Metabolomic GC/MS data usually requires some type of pre-processing before multiple sample comparisons and compound identifications can be carried out. This can be achieved by applying a methodology called curve resolution, or deconvolution, to the data. By the introduction of multivariate curve resolution (MCR)[16], multiple samples could be resolved to generate a common set of descriptors suitable for comparison using, for example, multivariate data analysis. A further development of MCR, done in our lab, named hierarchical-MCR (H-MCR)[21], allows complex GC/MS data, as generated within metabolomics, to be resolved into its pure components. An extension to the H-MCR method made it possible to perform the curve resolution predictively [22]. By combining the H-MCR processing with multivariate data analysis, a strategy is obtained for multivariate data processing and analysis, which is efficient for highlighting patterns of resolved and identified metabolites systematically co-varying over multiple samples [23,24,25]. This strategy is predictive in both the processing and modeling part, which makes it interesting for the development of high-throughput metabolomic screening, diagnostic systems, metabolite pattern verification over multiple studies or even for clinical use. In contrast to other processing methods, such as AMDIS [26], ChromaTOF (LECO, St. Joseph, MI), Tagfinder [27], and ADAP [28], H-MCR processes all or a subset of all samples together, while the other methods process one sample at the time, or in some cases simultaneously—although independently—using parallel computing. We believe that by processing all samples together, the outcome of the processing will be more suitable for multivariate sample comparison, since a) all metabolites are quantified in the same way, b) no missing values will appear and c) there is no need for matching of resolved/deconvoluted peaks. However, possible disadvantages can be that a) strongly deviating samples can degrade the processing outcome (can be solved by thoroughly selecting samples to base processing upon; samples that deviate due to analytical error should be excluded), b) metabolites that are present only in a single or a small portion of the samples might not be detected, especially if they are in low concentration and c) the data processing is memory-demanding in case of many samples. This is true if all samples are processed instead of using a representative subset.

In this paper, we show that by selecting representative sample subsets to generate a reference table with reliably quantified and identified metabolites, by means of H-MCR, and performing multivariate regression analysis, using orthogonal projections to latent structures discriminant analysis (OPLS-DA)[29,30], an efficient metabolomic analysis is attained for GC/TOFMS data on human blood serum samples. The samples were collected in a study of the effect of strenuous physical exercise in humans; 24 healthy and regularly training male subjects participated in four identical 90 minutes tests of strenuous ergometer cycling exercise. Blood samples were taken before and directly after each exercise session to generate insights into human metabolism in relation to acute physical exercise. We investigated how the suggested method can be used to address the issues of performing a reliable screening by selecting samples according to two different strategies, one based on metadata variables and the other based on already acquired GC/TOFMS data processed using a fast and crude processing method. These two strategies were developed to be applicable for sample bank mining and efficient screening of large sample sets. Both strategies were also used to exemplify the usefulness of the method as a diagnostic tool by predictively verifying a pattern of identified or identifiable metabolites in a set of human blood samples analytically characterized by GC/TOFMS eight months later than the model samples.

## 2. Results

**Figure 1:**
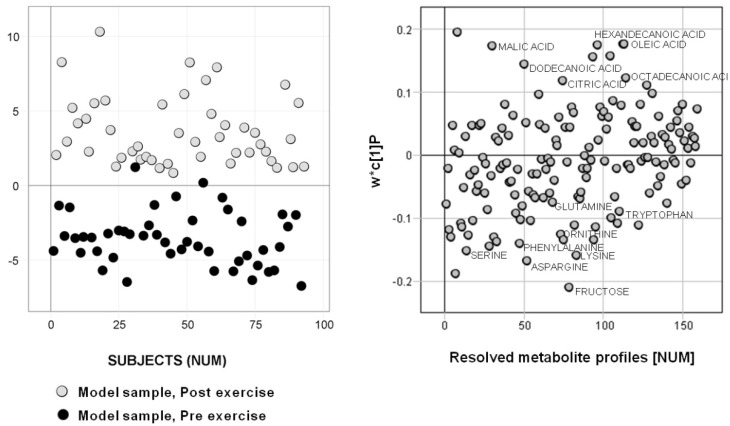
a) Classification model of the acute effect of strenuous exercise. Cross validated OPLS-DA score plot for the 93 model samples from exercise occasions one and two showing separation between pre- exercise (black circles) and post- exercise (gray circles) serum samples with a classification accuracy of 97.9% (class prediction (CV)). b) OPLS-DA covariance loading plot showing the variable (metabolite) contribution to the separation in the model scores. A selection of identified amino and fatty acids is named in the plot.

GC/TOFMS was used to metabolically characterize 96 plasma samples taken pre- and post- exercise from two separate test occasions (referred to as test occasions one and two). The resulting data were subjected to H-MCR processing to obtain a reliable quantification and identification of detected metabolites, *i.e.,* generation of a reference table of putative metabolites in the analyzed samples. The generated reference table was used for OPLS-DA classification modeling of the systematic metabolic variation related to the acute effect of strenuous exercise. Three samples were excluded prior to data processing and analysis due to insufficient derivatization, giving a total of 93 samples for further investigations. The H-MCR processing of the 93 samples resulted in reference table containing 167 resolved metabolites. The area under the resolved chromatographic profiles was used as sample descriptors in multiple sample comparison between samples taken pre- and post- exercise. The cross validated OPLS-DA score plot revealing the separation between pre- and post- exercise samples is shown in Figure 1a**,** and the resolved metabolite profiles responsible for the separation are shown in the corresponding covariance loading plot (Figure 1b). A general interpretation of the model loadings show a decrease of some amino acids in combination with elevated levels of fatty acids in the blood during exercise. These results can also be verified physiologically as amino acids enter pathways to sustain blood glucose homeostasis as a substrate to gluconeogenesis in the liver and kidneys, as well as oxidation in skeletal muscle. During exercise, fatty acids are released into circulation from adipose tissue and utilized as an energy substrate in working skeletal muscle, particularly during submaximal and prolonged exercise [31]. Identified metabolites in the study are listed in the supporting table S1, and the model parameters for the multivariate sample comparisons are listed in supporting table S2.

### 2.1. Subset Selection 1 — Metadata

A total of 34 metadata variables characterizing the 24 male subjects included in the study were subjected to PCA analysis. Two principal components were extracted describing 78.8% of the variation in the data (R2X = 0.788), and the resulting score vectors were used for a diversity-based selection of a representative sample subset. Six subjects were selected creating a subset of twelve samples (six subjects, pre- and post- exercise) that spanned the score space maximally. The acquired GC/TOFMS data for the selected subset was subjected to H-MCR processing resulting in a reference table containing 233 resolved putative metabolites. The area under the resolved chromatographic profiles were used as sample descriptors in OPLS-DA analysis for multiple sample comparison that revealed a clear separation between pre- and post- exercise samples in relation to metabolic composition. The metabolic information content in the subset was compared to the information content in the total dataset (obtained when applying H-MCR processing to all samples from exercise occasion one and two). The percentage of shared spectral metabolite profiles in the two reference tables was 87.4% (146/167). The percentage of shared metabolite profiles significantly separating pre- and post- exercise samples between the subset and the total data set, identified by a permutation test, was 94.1% (32/34). In addition, the remaining samples from test occasions one and two were predictively processed to detect and quantify the metabolites in the reference table, followed by predictive classification into the OPLS-DA model. This resulted in a cross-validated classification accuracy for the model samples (n = 24) of 100% (Class prediction (CV)) and a predictive classification accuracy of 97.1% (Class prediction (Test Set)) for the test samples (n = 69). The representative subset selection was evaluated by repeating the procedure above for three additional selections, where each subject was included in one subset only. The results are presented in the supporting information (Figure S1 and Tables S2, S3 and S4). 

### 2.2. Subset Selection 2 — Analytical Data

**Figure 2:**
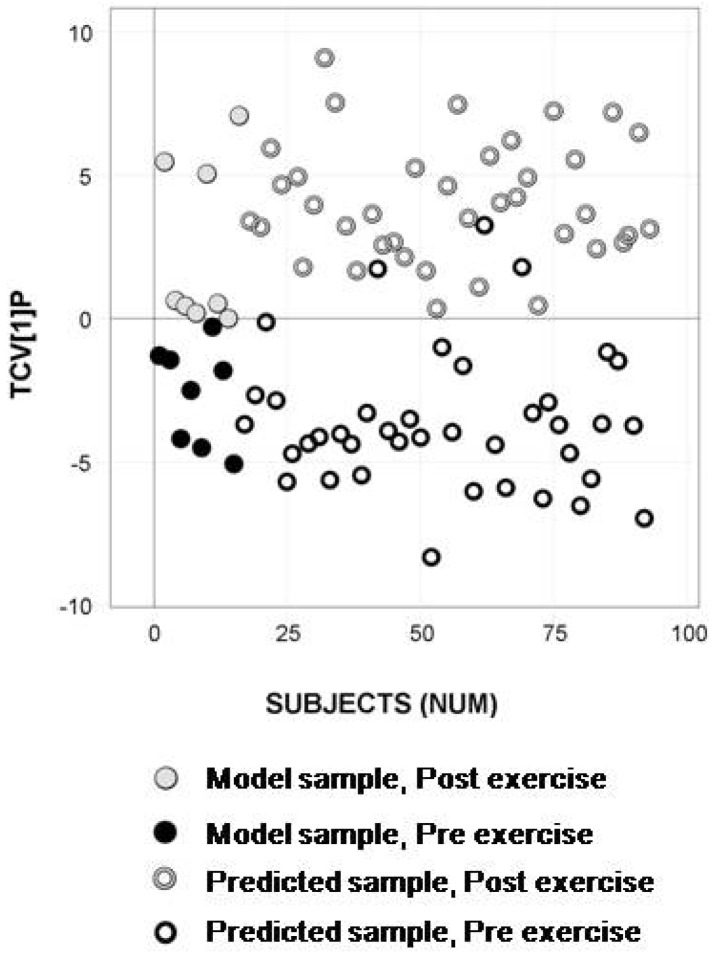
Classification model of the subset selected based on analytical data, including sample predictions. OPLS-DA predictive score plot showing separation between pre- exercise (black circles) and post- exercise (gray circles) serum samples with a cross-validated classification accuracy of 93.8% (Class prediction (CV)). Predictively classified test samples (remaining samples from test occasion one and two), marked as open circles, display high predictive classification accuracy (Class Prediction (Test set): 96.1%) for pre- exercise samples (black open circles) and post- exercise samples (gray open circles).

Human serum GC/TOFMS data of the 93 samples from exercise occasions one and two were processed using a fast processing method called hierarchical data compression [32]. The 230 resulting intensity vectors were used as descriptors in a PCA analysis of the pre- exercise samples. Three principal components were extracted describing 72.4% of the variation in the data (R2X = 0.724). A subject-wise subset selection was performed using a space-filling design in the PCA score space. Eight subjects were selected creating a set of 16 model samples, including pre- and post- exercise samples. The model samples were subjected to H-MCR processing, resulting in a reference table containing 168 resolved putative metabolites that were used as descriptors in the following multiple sample comparisons by means of OPLS-DA. The calculated OPLS-DA model revealed an evident separation between pre- and post- exercise samples in terms of metabolic composition (Figure 2). 

The metabolic information content in the model samples was compared to the information in the total dataset (obtained when applying H-MCR processing to all samples from exercise occasion one and two). The percentage of shared spectral metabolite profiles was 82.6% (138/167). The percentage of shared mass spectral metabolite profiles significantly separating pre- and post- exercise samples between the subset and the total data set, identified by the permutation test, was 88.2% (30/34). In addition, the remaining samples from test occasions one and two were predictively processed to detect and quantify the metabolites in the reference table, followed by predictive classification into the OPLS-DA model. This resulted in a cross-validated classification accuracy for the model samples (n=16) of 93.8% (Class prediction (CV)) and a predictive classification accuracy of 96.1% (Class prediction (Test Set)) for the test samples (n=77) (Figure 2). The time for H-MCR processing of the 16 selected samples was 6 h and 29 min, while predictive H-MCR processing of the remaining 77 test samples took only 10 min (<10sec/sample).

**Figure 3:**
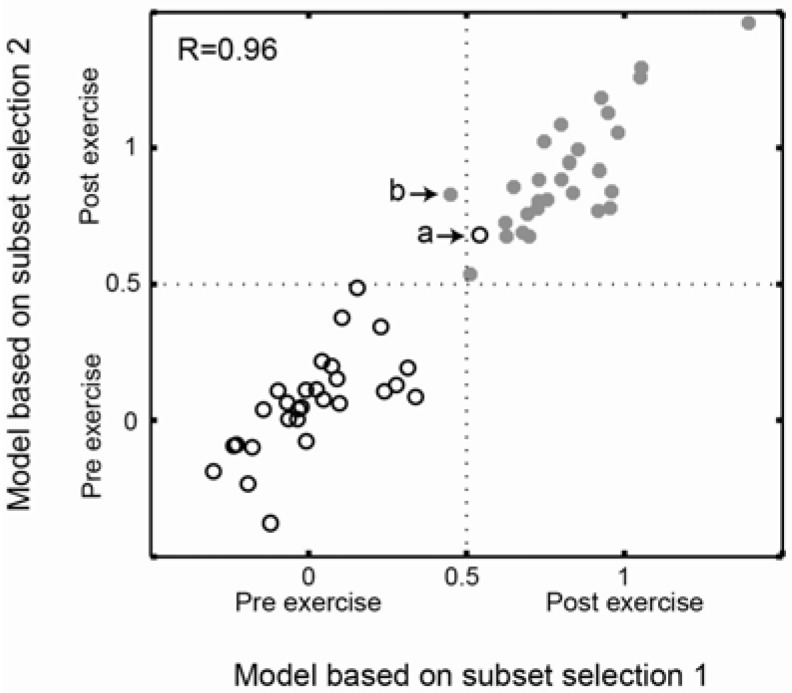
Comparison of prediction similarity for models based on the two subset selection strategies. The prediction values from the two models show a strong correlation, R=0.96 (Pearson correlation). This implies that both models did find the same or a similar metabolic pattern, differentiating the two study groups. All samples except one were classified equally by the two different models in terms of class belonging. One of the samples in the pre- exercise group were wrongly predicted by both models (marked a), while one of the post- exercise samples were predicted correctly by the subset selection 2, but wrongly by the subset selection 1(marked b).

### 2.3. Comparison of Prediction Similarity of Models Based on Subset Selections

In order to compare the predictive ability of the models generated by the two subset selection strategies, we formed a test set including the samples that were outside both selections. The test set, including 57 samples (29 pre- exercise (0) and 28 post- exercise (1)), were used to show the differences/similarities in prediction power for the two different models (subset selection 1-meta data and subset selection 2-analytical data) (Figure 3). 

### 2.4. Longitudinal Sample Predictions

**Figure 4:**
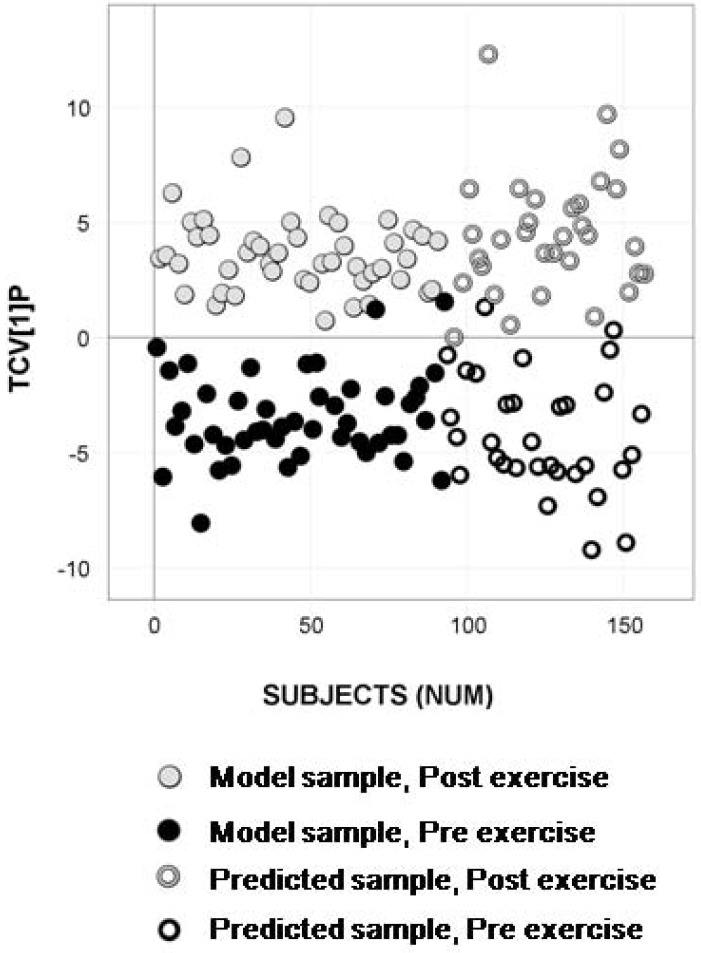
Longitudinal sample predictions in the classification model for subset selection 1- metadata. OPLS-DA predictive score plot of the model updated with the remaining samples from exercise occasion one and two showing separation between pre- exercise (black circles) and post- exercise (gray circles) serum samples (Class prediction (CV): 97.8%). Predicted test samples from exercise occasions three and four marked as open circles display high predictive classification accuracy (Class prediction (Test set): 96.9%) for pre- exercise samples (black open circles) and post- exercise samples (gray open circles).

**Figure 5:**
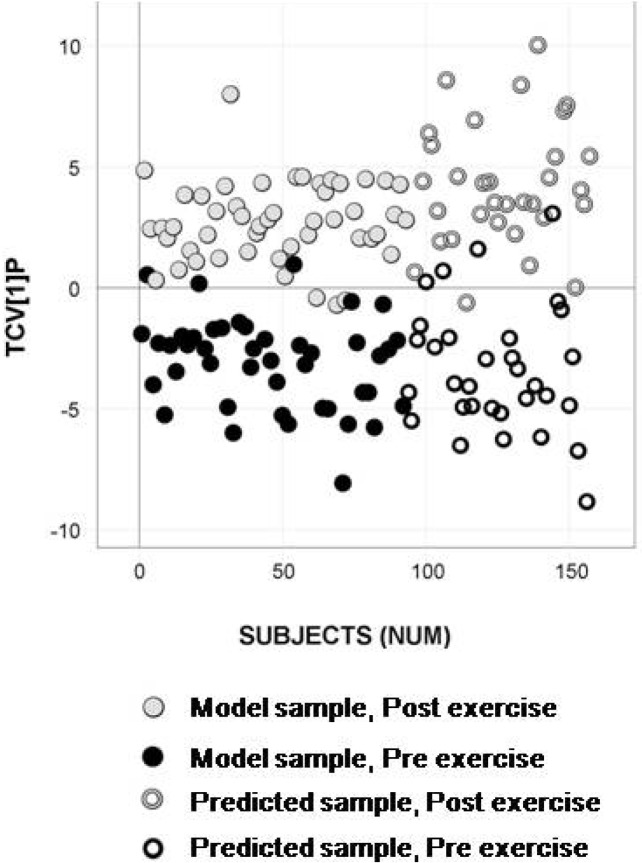
Longitudinal sample predictions in the classification model of subset selection 2 -analytical data. OPLS-DA predictive score plot of the model updated with the remaining samples from exercise occasion one and two showing separation between pre- exercise (black circles) and post- exercise (gray circles) serum samples (Class prediction (CV): 93.5%). Predicted test samples from exercise occasion three and four marked as open circles display high predictive classification accuracy (Class prediction (Test set): 92.2%) for pre- exercise samples (black open circles) and post- exercise samples (gray open circles).

**Figure 6:**
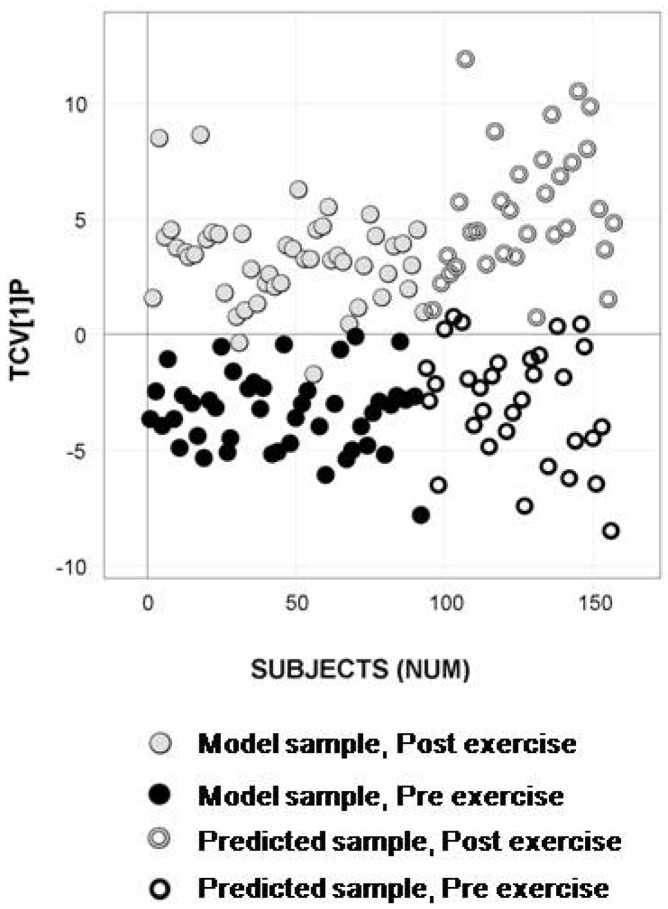
Longitudinal sample predictions in the classification model for the entire sample set. OPLS-DA predictive score plot for the model based on the 93 samples from exercise occasions one and two showing separation between pre- exercise (black circles) and post- exercise (gray circles) serum samples (Class prediction (CV): 97.8%). Predicted test samples from exercise occasions three and four marked as open circles display high predictive classification accuracy (Class prediction (Test set): 92.2%) for pre- exercise samples (black open circles) and post- exercise samples (gray open circles).

Samples from two additional exercise sessions (referred to as exercise occasions three and four) that were analytically characterized eight months later compared to the model samples were predictively processed to detect and quantify the metabolites in the reference tables. The updated OPLS-DA models based on significantly separating metabolic marker patterns, extracted using permutation tests, showed an evident separation between the samples taken pre- and post- exercise, in addition to a high predictive ability of the longitudinal samples (n = 64). This is shown for the OPLS-DA model based on the subset selected from metadata (Figure 4), the subset selected from acquired analytical data (Figure 5) and the model of the 93 samples from exercise occasions one and two (Figure 6). The prediction results for the subsets, as well as the results from the processing and modeling of all 93 samples concurrently, are listed in supporting table S4.

## 3. Discussion

### 3.1. Data Processing and Analysis

The results highlight that, by selecting a representative subset of samples, the metabolic information from, for example, a sample bank can be extracted and evaluated in a reliable fashion. Also, the predictive features of the strategy made it possible to process and classify new samples based on the information extracted from the selected subsets. This was shown by the fact that the H-MCR processing resolved a similar number of profiles (n = 206–233) for each of the four subsets selected based on the variation in metadata. OPLS-DA models based on each individual subset gave similar classification results, both in terms of cross validation (91.3%–100%) and predictive classification of the samples from the other three subsets (93%–97.1%). In addition, comparison of the results for the subset models to the results from when all samples were processed and modeled together showed that the subset models contained the same metabolic information as the model based on all samples

The method’s ability for efficient processing and classification of large data sets by selecting representative subsets was exemplified by the results based on the 16 samples selected from already acquired GC/MS data. In this case the predictive H-MCR processing and OPLS-DA classification was applied to the remaining samples in order to allow a high-throughput processing of many samples with retained data quality for interpretation and biomarker pattern identification. By using the selected subset to create a reference table of metabolites and predictively processed remaining samples to detect and quantify the metabolites in the reference table, an efficient strategy for screening of large data sets for producing representative and high quality data was offered. The proof for this was given by the prediction results for the OPLS model based on the subset, which correctly classified 96.1% of the remaining samples. Investigation of the metabolic information content revealed that 138 out of 167 metabolite profiles and 30 out of 34 metabolite profiles significantly discriminating the sample was detected in the reference table as compared to the data where all samples were resolved. This indicates that a small subset selected based on acquired GC/MS data, if done in a systematic fashion, will efficiently retain the variation in the original data. Here, the importance of a feasible sample selection approach that retains the systematic variation in the data must be highlighted. In high complexity data, such as the data presented here, this can be achieved by chemometric approaches, where selections are made in the compressed multivariate space, as offered by PCA for example. These results also highlight an interesting and important issue, namely that by selecting a representative subset of samples, significant metabolic information can be retrieved from a small number of samples, information by the presented method that can be predictively verified in follow up studies. This is important since it is not reasonable to believe that all future studies of value should include thousands of samples. Instead, there could be value in detecting potentially relevant information in small, well designed studies. The benefits of this will be many, including an efficient use of biobank samples, as well as better possibilities for maintaining a high analytical data quality, which is a major problem when analyzing large sample sets over longer times with mass spectrometry.

It is also worth noting that the same strategy as above, namely selecting representative sample subsets from acquired GC/MS data and utilizing the predictive feature of the H-MCR method, was used as an internal cross-validation procedure for the H-MCR curve resolution as a means to obtain a robust and reliable metabolite pattern on which to base further modeling and interpretations. Thus, the whole chain of events, including multivariate curve resolution and sample classification, is subject to an internal cross-validation procedure, as well as providing the possibility for prediction of new independent samples, which to our knowledge makes the proposed strategy unique.

Of high importance for an efficient screening of large sample sets is the time and feasibility for producing representative data. Curve resolution methods are, in general, very time-consuming and demanding in terms of computer capacity, which limits the number of samples that can actually be processed. However, the predictive feature of the H-MCR method can resolve this issue. In the given example, H-MCR processing of the 16 samples took 6h and 29 min to resolve, while predictive processing of the remaining 77 samples took <10sec/sample. This indicates that as long as the selected subset is representative in terms, retained variation large sample quantities can be efficiently processed, providing data of high quality for sample comparisons, biomarker detection and identification, and predictions. It should be pointed out that the number of samples used in this example cannot be considered as a particularly large sample or data set. However, the point was proven that samples could be efficiently processed, with retained data quality, based on a selected set of representative samples, and as far as the method goes, there is no limitation to the number of samples that can be predictively processed in the same way as shown here. This high-throughput property of the methodology makes it interesting as a contribution to the recently presented metabolome-wide association studies (MWAS), where the aim is to analyze several thousands of samples in the search for metabolic phenotypes that can aid in, for example, disease treatment or prevention [33,34]. MWAS have so far mainly been carried out with nuclear magnetic spectroscopy (NMR) as the analytical platform, mainly due to the high robustness, the simplicity in terms of sample handling and the speed of data processing in comparison to mass-spectrometry-based methods. However, the rapid development of instrumentation and automated sample preparation equipment for mass spectrometry have made it feasible to utilize the higher sensitivity and information content of mass spectrometry data for MWAS. In this, we see our presented data processing as a key component for efficiently generating high quality data for metabolic phenotyping in such large sample sets.

The ultimate goal for an efficient screening method in terms of a diagnostic is to perform in a robust and reliable fashion over time. We believe that the presented approach has the ability do so, however this is still something that needs to, and will be, evaluated in much more detail. As proof of the capability of the method in terms of providing correct classifications over time, predictions were made of samples from two exercise sessions that were analytically characterized eight months later compared to the model samples. Predictive processing and classification was carried out using all subset models, as well as all model samples. The results showed that, irrespective of subset model, a high classification accuracy was obtained for the new samples, and this accuracy was comparable to the one obtained when using all model samples for carrying out the predictions. The interpretation of this is that the method is efficient in carrying out longitudinal predictions based on a biomarker pattern extracted eight months earlier in time. Hence, we can conclude that the proposed method is showing promising results as a means for predictive screening in terms of biomarker pattern verification and diagnosis.

Although the presented strategy is promising, there are still challenges ahead in order to reach the stage where a complete and robust method for screening and predictions over time is in place. For instance, to further evaluate the strategy, it would be of value to perform a separate study applying the same test to a completely independent study population and make predictions for these subjects into the existing model. Also, this study was carried out on a homogenous human population, which is not representative for the whole human population, which is obviously something that needs to be considered, for example, in disease diagnosis modeling. One extremely important factor for these types of approaches to be successful will always be properly designed studies carried out according to a standardized protocol under as standardized conditions as possible, which is the case for the presented study. Dealing with human subjects, and that is eventually where many research projects are aiming today, there is a need to minimize confounding variation, or at least to be able to detect and handle this variation. In this, the chemometric methodology in terms of design of experiments and multivariate projections can bring a valuable contribution together with experience and knowledge related to the study itself [35]. Other issues that constantly need to be considered and optimized are standardization and quality control of sample handling and analytical characterization, as well as strategies for continuous updating of models to assure robust and reliable end results [20,36,37,38,39]. 

### 3.2. Biological Relevance

Interpreting the metabolite pattern reveals that the apparent increase in fatty acids in blood serum following exercise could be expected and does reflect an increased lipolysis and release of fatty acids from the adipose tissue. This is stimulated by catecholamines and other stress-induced hormones during exercise [40,41]. It is known that fatty acid metabolism increases in working muscle fibers and that this is related to the intensity and duration of exercise [42] together with training and muscle glycogen state [43,44]. Of the detected amino acids, aspargine, lysine, serine, phenylalanine, methionine, arginine, ornithine, proline, histidine, allothreonine, tryptophan, as well as the branched chain amino acids (BCAAs) valine and isoleucine, all decreased significantly (Figure 2) from pre- to post- exercise, while an increase in the level of alanine was seen at the same time. Many of these amino acids, particularly alanine, play a glucogenic role in hepatic glucose production, which does increase during exercise [45]. Thus, the release of alanine from skeletal muscle into blood may have exceeded uptake to the liver. Conversely, the decreased level of the other detected amino acids may be related to greater uptake and utilization in hepatic gluconeogenesis. As the utilization of amino acids, predominantly glutamate and the BCAAs, increases in muscle during prolonged exercise to support the muscle ATP-synthesis, a release of glucogenic amino acids from working muscles may be less than the hepatic uptake [46,47]. In addition, the increased level of inosine detected does reflect the well characterized adenine-nucleotide catabolism (ATP→ADP→AMP→IMP→inosine) that occurs in working muscle during strenuous exercise [48,49], and, consequently, an increased release of inosine from muscle to blood [50]. In summary, this proves that the generated models based on the detected and resolved metabolites do provide biologically relevant information, which of course is key to further application of the methodology in research, as well as for clinical applications.

## 4. Experimental Section

### 4.1. Dataset

24 healthy and regularly training male subjects (age: 25.7 ± 2.7 yr; height: 182.5 ± 7.6cm; bodyweight: 77.4 ± 8.8kg; VO_2peak_ at 59.1 ± 7.3mL kg^−1^min ^−1^) volunteered to participate in the study. The study was approved by the regional ethical committee (Dnr 05–069M). Each subject participated in a pre-experimental VO_2peak_ test and four identical experimental tests performed one week apart. Samples from two test occasions were used in the primary analysis, while samples from the remaining two test occasions were used for longitudinal predictions and for that reason, characterized analytically by GC/TOFMS eight months later. The dataset included in total 160 samples, *i.e.,* 96 samples used in the primary analysis (24 subjects at two occasions and two time points) and 64 additional samples characterized eight months later (12 subjects at two additional occasions and two time-points along with 16 analytical replicates). The data have been previously used for evaluating physiological variation related to the acute effect of strenuous exercise [51]. Raw data is available upon request.

#### 4.1.1. Pre-Experimental Procedures

The included subjects performed a pre-experiment incremental test on an ergometer cycle (Monark 839E) to exhaustion in order to determine the maximum oxygen uptake as a mean of 60 seconds (VO_2peak_) [52]. At the morning of the experimental test a standardized breakfast in amount related to bodyweight was ingested at 7.30 am, one hour prior to the test. Subjects were instructed to maintain food diaries prior to exercise occasion one and then repeat the same diet prior to exercise occasions two, three and four. Subjects were also instructed not to perform any exercise or consume alcohol the day before each exercise occasion and to avoid stress in the morning of the test day.

#### 4.1.2.Experimental Procedure

Venous blood samples were taken after 15 min of bed rest by using a vacutainer system (Becton Dickinson, UK). Thereafter, subjects were equipped with an intravenous catheter (Optiva^®^2, Medex) in a forearm vein, a transmitter belt (Polar WearLink^TM^31) and a heart frequency monitor Polar S610i^TM^). Subjects then performed 90 min of ergometer cycling, using an electronically braked bicycle (Rodby^TM^, RE 829, Enhörna, Sweden). Each 90 min test session consisted of nine equal 10 minutes sections. The workloads during the sections were loads that corresponded to 40% (2 min), 60% (6 min) and 85% (2 min) of the VO_2peak_ value from the pre-experimental test. 100ml of water was ingested after every 10 min of cycling. Immediately after 90 min completed cycling, blood was collected from the vein catheter into vacutainer tubes. Serum was extracted from the collected blood samples following 8 min centrifugation (+4 °C at 3000*g*) and immediately frozen and stored in -80 °C. Prior to GC/TOFMS analysis, the serum samples were extracted and derivatized according to A *et al.* [53] The samples were injected in splitless mode by an Agilent 7683 autosampler (Agilent, Atlanta, GA) into an Agilent 6890 gas chromatograph equipped with a 10 m x 0.18 mm i.d. fused silica capillary column with a chemically bonded 0.18 *µ*m DB 5-MS stationary phase (J&W Scientific, Folsom, CA). The column effluent was introduced into the ion source of a Pegasus III time-of-flight mass spectrometer, GC/TOFMS (Leco Corp., St Joseph, MI). Detailed description of the pre-experimental procedures, blood sampling, sample preparation, derivatization and GC/TOFMS protocol are found in the supporting text.

### 4.2.Selection of Representative Samples

Two alternatives for the selection of representative sample subsets for data processing were investigated; (1) to base the selection on metadata and (2) to base the selection on already acquired analytical data (GC/TOFMS). The selection was based on the systematic variation captured in the meta- or analytical data by principal component analysis (PCA). Each sample subsets was selected so that the systematic variation in the original set was maintained in the best possible way [54,55,56].

#### 4.2.1. Subset Selection 1— Metadata

The included human subjects were characterized by 34 metadata variables including age, weight, maximum pulse at pre-test, VO_2peak_, load at different percentage of VO_2peak_, serum glucose and hemoglobin levels (supporting table S5). The metadata variables were subjected to PCA and the inter-sample relationship was investigated for deviating observations before diversity-based selections were carried out. A subset was selected mimicking a representative selection of samples from a sample bank. The subset was separately analyzed by GC/TOFMS, resolved by means of H-MCR to obtain a reliable quantification and identification of detected metabolites, *i.e.,* a reference table of putative metabolites in the analyzed samples. The quantified metabolites in the reference table were analyzed by multivariate OPLS-DA classification modeling. The reference table based on the selected subset was then used to detect and quantify the metabolites in the in the remaining independently analyzed samples, *i.e.,* predictive processing.

#### 4.2.2. Subset Selection 2—Analytical data

Acquired GC/TOFMS data for all samples from test occasion one and two were subjected to hierarchical multivariate data compression [32], providing a fast and crude description of the compositional differences among the samples while retaining the systematic variation in the data. PCA was applied to the resulting intensity vector data. The inter-sample relationship was investigated for deviating observations before diversity-based selections were carried out. The selection was performed using a space-filling design which maximizes the minimum Euclidean distance between the nearest neighbors of the selected observations [57], thus maximizing the variation in all properties in the original space. Pre- and post- exercise samples corresponding to the selected subset were then resolved to create a metabolite reference table by means of H-MCR and multivariately classified using OPLS-DA. The reference table based on the selected subset was then used to detect and quantify the metabolites in the in the remaining samples, *i.e.,* predictive processing.

### 4.3. Generation, Processing and Modeling of Representative Data

#### 4.3.1. Data Processing and Analysis

GC/TOFMS data files were exported to MATLAB software (Mathworks, Natick, MA), where all data processing procedures and the space-filling design were performed using in-house scripts. The multivariate analysis was carried out in the SIMCA-P+ software (MKS Umetrics AB, Umeå, Sweden). NIST MS Search 2.0 (NIST, Gaithersburg, MD) was used for compound identification based on comparison between resolved spectra and standard spectra from NIST 08 mass spectra library, in-house mass spectra library or the MaxPlanck Institute mass spectra library (http://csbdb.mpimpgolm.mpg.de/csbdb/gmd/gmd.html).

#### 4.3.2. Hierarchical Multivariate Curve Resolution (H-MCR)

Multivariate Curve Resolution (MCR) [16] is a method for simultaneous resolving multiple GC/MS samples (X) into chromatographic (C) and spectral (S) profiles. MCR calculates a common spectral profile (S) (a mass spectrum) for each resolved profile and for each sample a corresponding chromatographic profile (C) is obtained and (E) is the residual consisting of instrumental noise and possible also unresolved components, see eqv 1. 


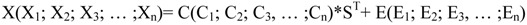
(1)

Due to the size and complexity of metabolic data, MCR decomposition of data into spectral and chromatographic profiles cannot be done for the complete data set simultaneously. To cope with this the data is divided into smaller parts. This is done in by splitting the data in the chromatographic dimension into a set of time window. Prior to this division, the samples are aligned in the chromatographic dimension. Each time window is then resolved separately using MCR. This procedure is called H-MCR [21]. For new independent samples chromatographic profiles (C) can be calculated using the common spectral profiles (S) using Equation (2). In this way, a new set of samples can be resolved predictively, meaning that the same set of profiles are obtained (the same metabolites are resolved)[22]. The collection of all spectral profiles from all time windows can be seen as a reference table of putative metabolites. 


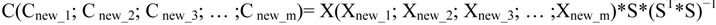
(2)

This predictive feature of MCR also made it possible to integrate an internal validation step in the processing. By dividing the samples to be resolved into two sets and performing independent resolution of the two sets, interchanging S_setA_, C_setA_, S_setB_ and C_setB_ are obtained. Samples in set 1 are then predicatively resolved using S_setB_ to get C_setA_pred_ and set 2 are then predictively resolved using S_setA_ to get C_setB_pred_. By comparing the similarity between S_setA_ and S_setB_, C_setA_ and C_setA_pred_ and C_setB_ and C_setB_pred_, respectively, it is possible to identify the profiles that are stable across samples. Here we use Pearson correlation above 0.95 as the criterion for stability. Only profiles that meet this criterion for all comparisons are used. In this way a reference table consisting of verified and stable spectral profiles is created. To ensure that the reference list is created using representative samples, the selection of “set A” and “set B” are made independently for each time window using principal component analysis (PCA) and space-filling design [57], where each sample is characterized by a total mass spectrum (sum of all mass spectrum (scans) in a time window). This dynamic selection of samples will enhance the metabolite coverage in each time window. 

#### 4.3.3. Multivariate Classification and Prediction

Prior to multivariate analysis, the data of all putative metabolites (integrated areas under the metabolites chromatographic profiles) were normalized using the weighted sum of the concentrations of 11 labeled internal standards (listed in supporting text) eluting over the whole chromatographic time range. OPLS-DA [29,30] was used to highlight patterns of metabolites that were systematically co-varying over multiple samples in relation to the acute effect of strenuous exercise and to investigate the robustness of these patterns. This was done by correlating the resolved metabolic information against the exercise phase (pre- *vs.* post- exercise) and predicting independent samples with known phase into existing models. Data were mean-centered and scaled to unit variance prior to modeling, and the number of significant OPLS-DA components was decided by seven-fold full cross validation [58]. OPLS is a PLS algorithm [59] with an integrated orthogonal signal correction (OSC) filter [60], which allows the systematic variation correlated to the response, in this case exercise phase, to be modeled in one predictive component and the systematic variation not related to the response in orthogonal components. In this way, the prediction results could be visualized in the predictive OPLS-DA score vector (t1[p]) and a facilitated interpretation of the metabolic patterns related to exercise phase was obtained in the corresponding OPLS-DA covariance loading vector (w*1[p]). This is crucial for the understanding of complex biological data and in particular for human data, where the inter-person variability can be extensive, and hence is likely to confound the interpretation if not separated from the information of interest. 

### 4.4. Evaluation of Data Processing and Modeling

The strategy of processing large sample sets by selecting representative subsets that capture the metabolic variation in the entire sample set was evaluated by comparing parameters descriptive for the multiple sample comparisons, metabolic information content and sample predictions. The results obtained for the two selected representative sample subsets were compared to the results obtained when processing and modeling all samples concurrently.

#### 4.4.1. Multiple Sample Comparisons

The similarity between the OPLS-DA models in terms of described metabolic variation was evaluated by the calculated model parameters R2X, R2Y and Q2, where R2X and R2Y correspond to the fraction of the variation in the resolved GC/TOFMS data (X) and the binary class identity variable (y; 0 = pre-exercise and 1 = post-exercise), respectively, explained by the extracted OPLS-DA components and Q2 corresponds to the fraction of the total variation in y predicted by the model, according to 7-fold full cross-validation [58].

#### 4.4.2. Metabolic information content

The metabolic information in the sample subsets was compared to the information present in the entire sample set by matching of resolved metabolite profiles. The reference table from the H-MCR processing of the entire sample set was compared to the attained reference table for the subsets and the spectral similarity was decided by comparing retention time and the match factor obtained in NIST MS Search 2.0 (NIST, Gaithersburg, MD). The factors range from 999 for a perfect match to zero for spectra having no peaks in common. Resolved mass spectral profiles were considered to be equivalent if the match factor was above 700 and the retention times differed less then ± 1 second. Subsequently, the percentage of the overall shared resolved spectral profiles in the reference tables was calculated. The metabolic information in the processed data was further assessed by extracting metabolite profiles that significantly separated the two exercise states (pre- or post- exercise) by a permutation test. In the permutation test, the y-vector (in this case a vector containing information about class identity (pre- or post- exercise)) was permuted randomly 10 000 times, and for every permutation, a OPLS model [59] was created between the resolved GC/TOFMS data and the permutated y-vector. Metabolites showing a stronger correlation to the y-vector in the original model, *i.e.,* variables with elevated OPLS weight values (w1-values), compared to the permuted y models were extracted, and the percentage of significantly separating metabolite profiles shared between the entire dataset and each subset was calculated. 

#### 4.4.3. Sample Predictions

The predictive ability of the multivariate models was investigated by the number of model samples that was correctly classified according to seven-fold cross validation (CV) (Class Prediction (CV)), as well as the number of independent samples (Test Set) predicted into the right class by the OPLS-DA model (Class Prediction (Test Set)). Samples in the Test Set are predictive both in the case of the resolving of metabolites H-MCR and the OPLS-DA classification.

#### 4.4.4. Longitudinal Sample Predictions

Additional samples from exercise occasions three and four (n = 64) were used to investigate the methods ability as a means for predictively verifying a detected metabolic marker pattern in longitudinal studies, *i.e.,* its potential as a diagnostic tool. Exercise occasions three and four were performed by the same male subjects in conjunction with the other tests, but the samples were characterized analytically by GC/TOFMS eight months later. The idea here was to use each subset to extract a significantly separating metabolic marker pattern using the previously described permutation test and verify the pattern by means of classification of the samples from the additional exercise occasions. This was done by first assigning a predicted class membership (pre- or post- exercise) to each sample from exercise occasions one and two, which was used to update the existing models. The samples from exercise occasions three and four were then predictively resolved, using the reference table for the model samples, and classified by prediction into the new OPLS-DA models based on the significantly separating metabolic marker patterns. 

## 5. Conclusions

We show that by using chemometric strategies for selecting representative sample subsets, H-MCR curve resolution and multivariate classification can be used to efficiently screen large metabolomics data or sample sets with retained data quality, or to retrieve significant metabolic information from smaller sample sets that can be verified over multiple studies. 

## Supplementary Files

Supplementary File 1 PDF-Document (PDF, 742 KB)
