# Supplementary material for: Validated and Predictive Processing of Gas Chromatography-Mass Spectrometry Based Metabolomics Data for Large Scale Screening Studies, Diagnostics and Metabolite Pattern Verification
Source: Metabolites. 2012 Oct 31;2(4):796–817. doi: 10.3390/metabo2040796 (PMC3901241; doi:10.3390/metabo2040796)
Supplement: Supplementary File 1 — PDF-Document (PDF, 742 KB) [file metabolites-02-00796-s001.pdf]

Supporting Text

Pre-experimental procedures

Prior to the experimental tests, subjects performed a pre-experiment incremental test on an ergometer cycle (Monark 839E) to exhaustion in order to determine VO_{2peak} as a mean of 60 seconds. The MetaMax II (CORTEX Biophysik GmbH, Leipzig, Germany) system, which previously has been validated in our laboratory (Larsson et al. 2004), was used for determination of VO_2. The initial workload of 150 W was increased with 40 W every 3 minute until volitional exhaustion and the cadence was maintained at 70 rpm during the whole test. A standardized breakfast was ingested at 7.30 am, one hour prior to every experimental test. The breakfast consisted of drinkable yoghurt in an amount related to bodyweight (0.5g carbohydrate/kg bodyweight). The amount of protein and fat in the yoghurt were 3 and 0.5 percentage by weight of yoghurt respectively. Subjects were instructed to maintain food diaries prior to test 1 and then repeat the same diet prior to the second test. Subjects were also instructed not to perform any exercise or consume alcohol the day before each test and to avoid stress in the morning of the test day.

Blood sampling

The venous blood samples were all collected into vacutainer tubes, one SST^{TM} II Advance tube (BD Diagnostics-Preanalytical Systems, UK), one K3E 15% 0.054ml (BD Vacutainer Systems, UK) and two 9NC 0.129M tubes (BD Vacutainer Systems, UK). Blood was collected before (pre exercise) and immediately after (post exercise) completed ergometer cycling.

Sampling, extraction and derivatization of human blood serum samples

The metabolites were extracted with an extraction solution containing methanol and water (8:1). Eleven stable isotopic reference compounds representing different kinds of metabolites ([$^2H_4$]-succinic acid, [$^{13}C_5$,^{15}N]-glutamic acid, [$^2H_7$]-cholesterol, [$^{13}C_3$]-myristic acid, [$^{13}C_4$]-α-ketoglutarate, [$^{13}C_{12}$]-sucrose, [$^{13}C_4$]-hexadecanoic acid, [$^{13}C_5$]-Proline, [$^2H_6$]-salicylic acid, [$^2H_4$]-putrescine and [$^{13}C_6$]-glucose, 7 ng/ µL) were included in the extraction solution as internal standards. Serum (100µL) was mixed with extraction solution (900 µL). This mix was kept on ice for 10 min and extracted using a MM302 vibration mill (Retsch Gmb H & Co. KG, Haan, Germany) at 30 Hz for 2 min with a 3-mm tungsten carbide bead (Retsch GmbH & Co. KG). After extraction the samples were kept on ice for 2 h followed by 10 min centrifugation at 4° C (20,800 x g) and supernatants collected. As many serum metabolites are non-volatile, the samples were derivatized before GC/MS analysis. Therefore, 200 µL of the supernatant was transferred to a GC-vial and evaporated to dryness in a vacuum centrifuge. Derivatization was carried out in two

steps. First, 30 μL methoxyamine hydrochloride (15 mg/ mL) in pyridine was added and the vials were shaken vigorously for 12 minutes followed by 1 h incubation at 70 ºC and another 16 h at room temperature. Then, 30 μL of *N*-methyl-*N*-(trimethylsilyl)-trifluoroacetamide (MSTFA) with 1% trimethylsilyl (TMS) was added to each vial. The vials were shaken and incubated for 1 hour at room temperature. Just prior to GC-MS analysis 30 μL heptane, containing 15 ng/ μL methylsterate as an injection internal standard was added.

GC/MS. 1 μL of the derivatized sample was injected splitless by an Agilent 7683 autosampler (Agilent, Atlanta; GA, USA) into an Agilent 6890 gas chromatograph equipped with a 10 m x 0.18 mm i.d. fused silica capillary column with a chemically bonded 0.18 μm DB 5-MS stationary phase (J&W Scientific, Folsom, CA, USA). The injector temperature was 270°C, the septum purge flow was 20 ml min^{-1} and the purge was turned on after 60 s. The gas flow rate through the column was 1 ml min^{-1}, the column temperature was held at 70°C for 2 minutes, then increased by 40°C min^{-1} to 320°C, and held there for 1 min. The column effluent was introduced into the ion source of a Pegasus III time-of-flight mass spectrometer, GC/TOFMS (Leco Corp., St Joseph, MI, USA). The transfer line and the ion source temperatures were 250°C and 200°C, respectively. Ions were generated by a 70 eV electron beam at an ionization current of 2.0 mA, and 30 spectra s^{-1} were recorded in the mass range 60 to 800 m/z. The acceleration voltage was turned on after a solvent delay of 170 s.

Supporting table S1 – Identified metabolites

NAME	ID	MATCH VALUE [a]	RI-RI library
3-Amino-2-piperidone	HMDB00323	946	0
3-hydroxybutanoic acid	HMDB00357	861	8
Adenine	HMDB00034	773	-10
Adenosine-5-monophosphate	HMDB00045	781	4
alpha-Tocopherol	HMDB01893	897	2*
Arachidonic acid	HMDB01043	864	-3
Arginine	HMDB00517	924	-1
Asparagine	HMDB00168	940	0
Aspartic acid	HMDB00191	888	-7
beta-Alanine	HMDB00056	788	2
beta-D-Methylglucopyranoside	CAS 709-50-2	950	4
beta-Sitosterol	HMDB00852	726	2
Campesterol	HMDB02869	739	-10
Capric acid	HMDB00511	716	1*
Cholesterol	HMDB00067	910	8
Citric acid	HMDB00094	973	-3
Creatinine	HMDB00562	909	-2
Cysteine	HMDB00574	948	1
Cystine	HMDB00192	917	0
Docosahexaenoic acid	HMDB02183	889	2*
Dodecanoic acid	HMDB00638	843	-1
Elaidic acid	HMDB00573	942	1*
Erythrose	HMDB02649	643	-4
Fructose	HMDB00660	947	-5
Galactono-1,4-lactone	HMDB02541	933	3
Glucose	HMDB00122	952	-6
Glutamic acid	HMDB00148	882	0*
Glutamine	HMDB00641	960	0
Glyceric acid	HMDB00139	753	-2
Glycerol-3-phosphate	HMDB00126	920	-4
Glycine	HMDB00123	945	1*
Heptadecanoic acid	HMDB02259	632	-2
Heptanoic acid	HMDB00666	900	4
Histidine	HMDB00177	865	4
Hydroxyproline	HMDB00725	834	-2
Inosine	HMDB00195	790	-18
Isoleucine	HMDB00172	929	1*
Ketoleucine	HMDB00762	749	0*
Kynurenine	HMDB00684	690	-1
Linoleic acid	HMDB00673	937	-2
Lysine	HMDB00182	932	3
Malic acid	HMDB00156	901	-3
Methionine	HMDB00696	861	0

Methyl palmitate	CAS 112-39-0	745	0
myo-Inositol	HMDB00211	977	-3
myo-Inositol-1-phosphate	HMDB00213	923	-3
Nonanoic acid	HMDB00847	665	-3
Oleic acid	HMDB00207	824	-2
Ornithine	HMDB00214	946	-3
Palmitic acid	HMDB00220	925	12*
Palmitoleic acid	HMDB03229	935	-4
Phenylalanine	HMDB00159	950	0
Phosphoric acid	HMDB02142	868	-4*
Proline	HMDB00162	835	-4
Putrescine	HMDB01414	787	-4*
Pyroglutamic acid	HMDB00267	939	1
Quinic acid	HMDB03072	683	0
Stearic acid	HMDB00827	956	-6
Succinic acid	HMDB00254	865	0*
Taurine	HMDB00251	876	-4
Threonic acid	HMDB00943	947	-4
Threonine	HMDB00167	957	0*
Tryptophan	HMDB00929	961	2
Tyrosine	HMDB00158	958	1
Urea	HMDB00294	956	12
Uric acid	HMDB00289	904	1
Uridine	HMDB00296	824	-3

*RT-RT library

[a] Mass spectra match values according to NIST MS-Search 2.0.

Monosaccharidesare not included in the list with the exception of Fructose and Glucose

Supporting table S2 - Summary of descriptive parameters for OPLS-DA models

H-MCR RESOLVED, OPLS-DA MODEL SAMPLES	R2X (%)	R2Y (%)	Q2 (%)	CV-ANOVA (p-value)	OPLS COMPONENTS (NUM)	RESOLVED METABOLITES (NUM)	SAMPLES (NUM)
S1	31,7	92,6	76,4	3,5E-5	1+1	206	23
S2	37,0	98,7	87,0	1,1E-6	1+2	223	24
S3	19,9	96,6	78,0	2,0E-5	1+1	218	22
S4	40,4	97,8	84,7	4,3E-6	1+2	233	24
S5	30,7	90,9	52,2	0,067	1+1	168	16
S1-S4	25,1	83,7	75,4	5,7E-26	1+1	167	93

Abbreviations

S1 – Subset 1 based on property data

S2 – Subset 2 based on property data

S3 – Subset 3 based on property data

S4 – Subset 4 based on property data

S1-S4 – All pre- and post exercise samples from exercise occasion one and two.

S5 – Subset based on acquired analytical data

S6 – Test set for S5 i.e. remaining pre- and post exercise samples from exercise occasion one and two.

Supporting figure S1 - Representative subset selection based on property data

PCA score plots for the 24 subjects based on 34 property data variables used for diversity-based subset selection. The figure show each of the four subgroups (S1, S2, S3 and S4), and their location in the score space.

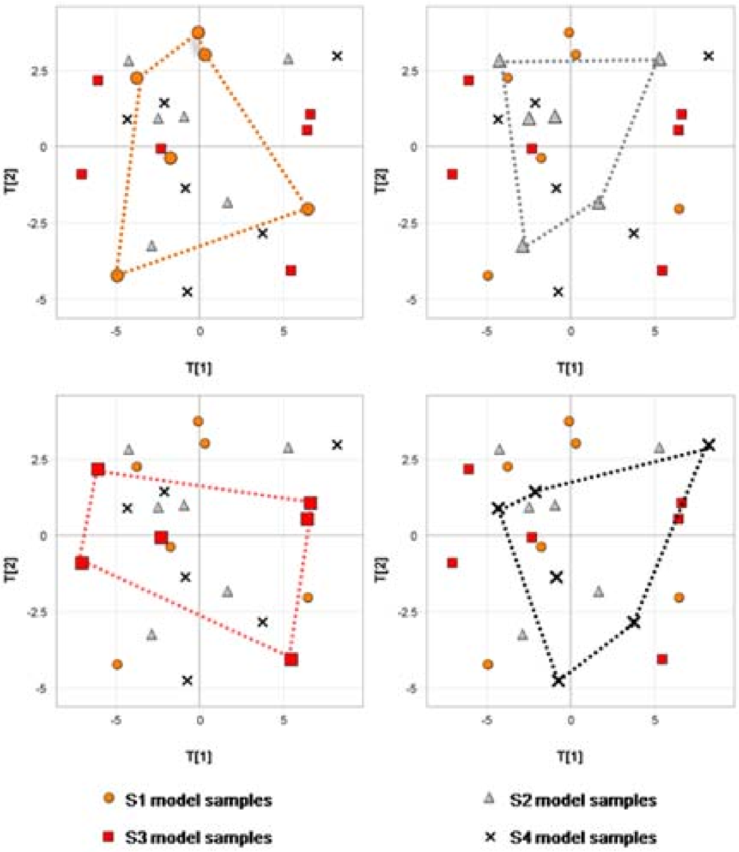

Supporting table S3 - Summary of sample prediction results

H-MCR RESOLVED, OPLS-DA MODEL SAMPLES	H-MCR PREDICTED, OPLS-DA TEST SAMPLES	CLASS PREDICTION (CV) (%)	CLASS PREDICTION (TEST SET) (%)
S1	S2, S3, S4	91,3	94,3
S2	S1, S3, S4	100	97,1
S3	S1, S2, S4	100	93,0
S4	S1, S2, S3	100	97,1
S5	S6	93,8	96,1
S1-S4	—	97,9	—

Abbreviations

S1 – Subset 1 based on property data

S2 – Subset 2 based on property data

S3 – Subset 3 based on property data

S4 – Subset 4 based on property data

S1-S4 – All pre- and post exercise samples from exercise occasion one and two.

S5 – Subset based on acquired analytical data

S6 – Test set for S5 i.e. remaining pre- and post exercise samples from exercise occasion one and two.

Table S4 Summary of longitudinal sample prediction results

H-MCR RESOLVED*, OPLS-DA MODEL SAMPLES	H-MCR PREDICTED, OPLS-DA TEST SAMPLES (n=64)	CLASS PREDICTION (CV) (%)	CLASS PREDICTION (TEST SET) (%)
**S1**+ S2, S3, S4	S7	97,8	92,2
**S2**+ S1, S3, S4	S7	95,7	92,2
**S3**+ S1, S2, S4	S7	91,4	93,8
**S4**+ S1, S2, S3	S7	97,8	96,9
**S5**+S6	S7	93,5	92,2
**S1-S4**	S7	97,8	92,2

*Bold text – H-MCR resolved samples

Abbreviations

S1 – Subset 1 based on property data

S2 – Subset 2 based on property data

S3 – Subset 3 based on property data

S4 – Subset 4 based on property data

S1-S4 – All pre- and post exercise samples from exercise occasion one and two.

S5 – Subset based on acquired analytical data

S6 – Test set for S5 i.e. remaining pre- and post exercise samples from exercise occasion one and two.

S7 - Samples from test occasion three and four analytically characterised 8 month after S1-S6

Supporting table S5 - metadata

	Subject 1	Subject 2	Subject 3	Subject 4	Subject 5	Subject 6	Subject 7	Subject 8	Subject 9	Subject 10	Subject 11	Subject 12	Subject 13	Subject 14	Subject 15	Subject 16	Subject 17	Subject 18	Subject 19	Subject 20	Subject 21	Subject 22	Subject 23	Subject 24
Subgroup	[illegible]	[illegible]	[illegible]	[illegible]	[illegible]	[illegible]	[illegible]	[illegible]	[illegible]	[illegible]	[illegible]	[illegible]	[illegible]	[illegible]	[illegible]	[illegible]	[illegible]	[illegible]	[illegible]	[illegible]	[illegible]	[illegible]	[illegible]	[illegible]
Hb (pre test 1)	[illegible]	[illegible]	[illegible]	[illegible]	[illegible]	[illegible]	[illegible]	[illegible]	[illegible]	[illegible]	[illegible]	[illegible]	[illegible]	[illegible]	[illegible]	[illegible]	[illegible]	[illegible]	[illegible]	[illegible]	[illegible]	[illegible]	[illegible]	[illegible]
Hb (0 min test 1)	[illegible]	[illegible]	[illegible]	[illegible]	[illegible]	[illegible]	[illegible]	[illegible]	[illegible]	[illegible]	[illegible]	[illegible]	[illegible]	[illegible]	[illegible]	[illegible]	[illegible]	[illegible]	[illegible]	[illegible]	[illegible]	[illegible]	[illegible]	[illegible]
Hb (15 min test 1)	[illegible]	[illegible]	[illegible]	[illegible]	[illegible]	[illegible]	[illegible]	[illegible]	[illegible]	[illegible]	[illegible]	[illegible]	[illegible]	[illegible]	[illegible]	[illegible]	[illegible]	[illegible]	[illegible]	[illegible]	[illegible]	[illegible]	[illegible]	[illegible]
Hb (30 min test 1)	[illegible]	[illegible]	[illegible]	[illegible]	[illegible]	[illegible]	[illegible]	[illegible]	[illegible]	[illegible]	[illegible]	[illegible]	[illegible]	[illegible]	[illegible]	[illegible]	[illegible]	[illegible]	[illegible]	[illegible]	[illegible]	[illegible]	[illegible]	[illegible]
Hb (60 min test 1)	[illegible]	[illegible]	[illegible]	[illegible]	[illegible]	[illegible]	[illegible]	[illegible]	[illegible]	[illegible]	[illegible]	[illegible]	[illegible]	[illegible]	[illegible]	[illegible]	[illegible]	[illegible]	[illegible]	[illegible]	[illegible]	[illegible]	[illegible]	[illegible]
Hb (90 min test 1)	[illegible]	[illegible]	[illegible]	[illegible]	[illegible]	[illegible]	[illegible]	[illegible]	[illegible]	[illegible]	[illegible]	[illegible]	[illegible]	[illegible]	[illegible]	[illegible]	[illegible]	[illegible]	[illegible]	[illegible]	[illegible]	[illegible]	[illegible]	[illegible]
Hb (pre test 2)	[illegible]	[illegible]	[illegible]	[illegible]	[illegible]	[illegible]	[illegible]	[illegible]	[illegible]	[illegible]	[illegible]	[illegible]	[illegible]	[illegible]	[illegible]	[illegible]	[illegible]	[illegible]	[illegible]	[illegible]	[illegible]	[illegible]	[illegible]	[illegible]
Hb (0 min test 2)	[illegible]	[illegible]	[illegible]	[illegible]	[illegible]	[illegible]	[illegible]	[illegible]	[illegible]	[illegible]	[illegible]	[illegible]	[illegible]	[illegible]	[illegible]	[illegible]	[illegible]	[illegible]	[illegible]	[illegible]	[illegible]	[illegible]	[illegible]	[illegible]
Hb (15 min test 2)	[illegible]	[illegible]	[illegible]	[illegible]	[illegible]	[illegible]	[illegible]	[illegible]	[illegible]	[illegible]	[illegible]	[illegible]	[illegible]	[illegible]	[illegible]	[illegible]	[illegible]	[illegible]	[illegible]	[illegible]	[illegible]	[illegible]	[illegible]	[illegible]
Hb (30 min test 2)	[illegible]	[illegible]	[illegible]	[illegible]	[illegible]	[illegible]	[illegible]	[illegible]	[illegible]	[illegible]	[illegible]	[illegible]	[illegible]	[illegible]	[illegible]	[illegible]	[illegible]	[illegible]	[illegible]	[illegible]	[illegible]	[illegible]	[illegible]	[illegible]
Hb (60 min test 2)	[illegible]	[illegible]	[illegible]	[illegible]	[illegible]	[illegible]	[illegible]	[illegible]	[illegible]	[illegible]	[illegible]	[illegible]	[illegible]	[illegible]	[illegible]	[illegible]	[illegible]	[illegible]	[illegible]	[illegible]	[illegible]	[illegible]	[illegible]	[illegible]
Hb (90 min test 2)	[illegible]	[illegible]	[illegible]	[illegible]	[illegible]	[illegible]	[illegible]	[illegible]	[illegible]	[illegible]	[illegible]	[illegible]	[illegible]	[illegible]	[illegible]	[illegible]	[illegible]	[illegible]	[illegible]	[illegible]	[illegible]	[illegible]	[illegible]	[illegible]
Hb (pre test 3)	[illegible]	[illegible]	[illegible]	[illegible]	[illegible]	[illegible]	[illegible]	[illegible]	[illegible]	[illegible]	[illegible]	[illegible]	[illegible]	[illegible]	[illegible]	[illegible]	[illegible]	[illegible]	[illegible]	[illegible]	[illegible]	[illegible]	[illegible]	[illegible]
Hb (0 min test 3)	[illegible]	[illegible]	[illegible]	[illegible]	[illegible]	[illegible]	[illegible]	[illegible]	[illegible]	[illegible]	[illegible]	[illegible]	[illegible]	[illegible]	[illegible]	[illegible]	[illegible]	[illegible]	[illegible]	[illegible]	[illegible]	[illegible]	[illegible]	[illegible]
Hb (15 min test 3)	[illegible]	[illegible]	[illegible]	[illegible]	[illegible]	[illegible]	[illegible]	[illegible]	[illegible]	[illegible]	[illegible]	[illegible]	[illegible]	[illegible]	[illegible]	[illegible]	[illegible]	[illegible]	[illegible]	[illegible]	[illegible]	[illegible]	[illegible]	[illegible]
Hb (30 min test 3)	[illegible]	[illegible]	[illegible]	[illegible]	[illegible]	[illegible]	[illegible]	[illegible]	[illegible]	[illegible]	[illegible]	[illegible]	[illegible]	[illegible]	[illegible]	[illegible]	[illegible]	[illegible]	[illegible]	[illegible]	[illegible]	[illegible]	[illegible]	[illegible]
Hb (60 min test 3)	[illegible]	[illegible]	[illegible]	[illegible]	[illegible]	[illegible]	[illegible]	[illegible]	[illegible]	[illegible]	[illegible]	[illegible]	[illegible]	[illegible]	[illegible]	[illegible]	[illegible]	[illegible]	[illegible]	[illegible]	[illegible]	[illegible]	[illegible]	[illegible]
Hb (90 min test 3)	[illegible]	[illegible]	[illegible]	[illegible]	[illegible]	[illegible]	[illegible]	[illegible]	[illegible]	[illegible]	[illegible]	[illegible]	[illegible]	[illegible]	[illegible]	[illegible]	[illegible]	[illegible]	[illegible]	[illegible]	[illegible]	[illegible]	[illegible]	[illegible]
Hb (pre test 4)	[illegible]	[illegible]	[illegible]	[illegible]	[illegible]	[illegible]	[illegible]	[illegible]	[illegible]	[illegible]	[illegible]	[illegible]	[illegible]	[illegible]	[illegible]	[illegible]	[illegible]	[illegible]	[illegible]		[illegible]	[illegible]	[illegible]	[illegible]
Hb (0 min test 4)	[illegible]	[illegible]	[illegible]	[illegible]	[illegible]	[illegible]	[illegible]	[illegible]	[illegible]	[illegible]	[illegible]	[illegible]	[illegible]	[illegible]	[illegible]	[illegible]	[illegible]	[illegible]	[illegible]		[illegible]	[illegible]	[illegible]	[illegible]
Hb (15 min test 4)	[illegible]	[illegible]	[illegible]	[illegible]	[illegible]	[illegible]	[illegible]	[illegible]	[illegible]	[illegible]	[illegible]	[illegible]	[illegible]	[illegible]	[illegible]	[illegible]	[illegible]	[illegible]	[illegible]		[illegible]	[illegible]	[illegible]	[illegible]
Hb (30 min test 4)	[illegible]	[illegible]	[illegible]	[illegible]	[illegible]	[illegible]	[illegible]	[illegible]	[illegible]	[illegible]	[illegible]	[illegible]	[illegible]	[illegible]	[illegible]	[illegible]	[illegible]	[illegible]	[illegible]		[illegible]	[illegible]	[illegible]	[illegible]
Hb (60 min test 4)	[illegible]	[illegible]	[illegible]	[illegible]	[illegible]	[illegible]	[illegible]	[illegible]	[illegible]	[illegible]	[illegible]	[illegible]	[illegible]	[illegible]	[illegible]	[illegible]	[illegible]	[illegible]	[illegible]		[illegible]	[illegible]	[illegible]	[illegible]
Hb (90 min test 4)	[illegible]	[illegible]	[illegible]	[illegible]	[illegible]	[illegible]	[illegible]	[illegible]	[illegible]	[illegible]	[illegible]	[illegible]	[illegible]	[illegible]	[illegible]	[illegible]	[illegible]	[illegible]	[illegible]		[illegible]	[illegible]	[illegible]	[illegible]
Age	[illegible]	[illegible]	[illegible]	[illegible]	[illegible]	[illegible]	[illegible]	[illegible]	[illegible]	[illegible]	[illegible]	[illegible]	[illegible]	[illegible]	[illegible]	[illegible]	[illegible]	[illegible]	[illegible]	[illegible]	[illegible]	[illegible]	[illegible]	[illegible]
$VO_{2,peak}$ (ml/min)	[illegible]	[illegible]	[illegible]	[illegible]	[illegible]	[illegible]	[illegible]	[illegible]	[illegible]	[illegible]	[illegible]	[illegible]	[illegible]	[illegible]	[illegible]	[illegible]	[illegible]	[illegible]	[illegible]	[illegible]	[illegible]	[illegible]	[illegible]	[illegible]
$VO_{2,peak}$ (ml/kg/min)	[illegible]	[illegible]	[illegible]	[illegible]	[illegible]	[illegible]	[illegible]	[illegible]	[illegible]	[illegible]	[illegible]	[illegible]	[illegible]	[illegible]	[illegible]	[illegible]	[illegible]	[illegible]	[illegible]	[illegible]	[illegible]	[illegible]	[illegible]	[illegible]
Workload at $VO_{2,peak}$	[illegible]	[illegible]	[illegible]	[illegible]	[illegible]	[illegible]	[illegible]	[illegible]	[illegible]	[illegible]	[illegible]	[illegible]	[illegible]	[illegible]	[illegible]	[illegible]	[illegible]	[illegible]	[illegible]	[illegible]	[illegible]	[illegible]	[illegible]	[illegible]
Workload 85% of $VO_{2,peak}$	[illegible]	[illegible]	[illegible]	[illegible]	[illegible]	[illegible]	[illegible]	[illegible]	[illegible]	[illegible]	[illegible]	[illegible]	[illegible]	[illegible]	[illegible]	[illegible]	[illegible]	[illegible]	[illegible]	[illegible]	[illegible]	[illegible]	[illegible]	[illegible]
Workload 60% of $VO_{2,peak}$	[illegible]	[illegible]	[illegible]	[illegible]	[illegible]	[illegible]	[illegible]	[illegible]	[illegible]	[illegible]	[illegible]	[illegible]	[illegible]	[illegible]	[illegible]	[illegible]	[illegible]	[illegible]	[illegible]	[illegible]	[illegible]	[illegible]	[illegible]	[illegible]
Workload 40% of $VO_{2,peak}$	[illegible]	[illegible]	[illegible]	[illegible]	[illegible]	[illegible]	[illegible]	[illegible]	[illegible]	[illegible]	[illegible]	[illegible]	[illegible]	[illegible]	[illegible]	[illegible]	[illegible]	[illegible]	[illegible]	[illegible]	[illegible]	[illegible]	[illegible]	[illegible]
Highest pulse at pre experimental test	[illegible]	[illegible]	[illegible]	[illegible]	[illegible]	[illegible]	[illegible]	[illegible]	[illegible]	[illegible]	[illegible]	[illegible]	[illegible]	[illegible]	[illegible]	[illegible]	[illegible]	[illegible]	[illegible]	[illegible]	[illegible]	[illegible]	[illegible]	[illegible]
Average watt/kg bodyweight	[illegible]	[illegible]	[illegible]	[illegible]	[illegible]	[illegible]	[illegible]	[illegible]	[illegible]	[illegible]	[illegible]	[illegible]	[illegible]	[illegible]	[illegible]	[illegible]	[illegible]	[illegible]	[illegible]	[illegible]	[illegible]	[illegible]	[illegible]	[illegible]
BMI	[illegible]	[illegible]		[illegible]		[illegible]	[illegible]	[illegible]	[illegible]	[illegible]	[illegible]	[illegible]	[illegible]		[illegible]	[illegible]	[illegible]	[illegible]	[illegible]	[illegible]	[illegible]	[illegible]	[illegible]	[illegible]
